# Computed tomography in pediatric blunt abdominal trauma: current evidence, challenges, and future directions — a systematic review and meta-analysis

**DOI:** 10.1186/s13049-026-01578-5

**Published:** 2026-02-07

**Authors:** Mohammed Alsabri, Shree Rath, Mohamed Amr Elkarargy, Amira A. Aboali, Ahmed Bostamy Elsnhory, Mohamed Hany Ezz, Eric Lusinski

**Affiliations:** 1 Emergency Department, Althawara Modern General Hospital, Sanaa, Yemen; 2 Pediatric Emergency Department, Department of Pediatrics, St. Christopher’s Hospital for Children, Philadelphia, PA USA; 3 Department of Pediatrics, All India Institute of Medical Sciences, Bhubaneswar, India; 4 School of Medicine, University College Cork, Cork, Ireland; 5 Department of Diagnostic and Interventional Radiology, Damanhour Teaching Hospital, General Organization for Teaching Hospitals and Institutes, Damanhour, Egypt; 6 Faculty of Medicine, Al-Azhar University, Cairo, Egypt; 7 Faculty of Medicine, Tanta University, Gharbiya, Egypt; 8 Pediatric Emergency Medicine Physician, EMS Medical Command Facility Director, Medical Director of Transport St. Christopher’s Hospital for Children, Philadelphia, PA USA

**Keywords:** Intra-abdominal injuries, Blunt trauma, Pediatrics, Computed tomography, Imaging, Meta-analysis

## Abstract

**Introduction:**

Assessment of intra-abdominal injuries (IAIs) in children is challenging due to unreliable physical examination, communication barriers, and the serious consequences of missed injuries. Computed tomography (CT) is widely used for its high sensitivity, but concerns persist regarding radiation exposure and resource utilization. This systematic review and meta-analysis aimed to quantify IAI prevalence, describe organ-specific injury patterns, and evaluate intervention and mortality outcomes in children with blunt abdominal trauma assessed using CT.

**Methods:**

We searched PubMed, Web of Science, Cochrane Library, and Scopus through August 2025 for studies evaluating pediatric blunt abdominal trauma, CT-based assessment, and CT-detected IAIs. Bayesian random-effects meta-analyses were used to estimate pooled prevalence and outcomes, with additional hierarchical and meta-regression models for organ-specific injuries and study-level covariates. Certainty of evidence was assessed using the GRADE framework.

**Results:**

Fifteen studies including 7,430 children were analyzed. The pooled posterior median prevalence of IAI was 84.5% (95% credible interval [CrI]: 62–94%), while the probability of intervention was 7.7% and mortality was 1.4%. Solid organ injuries predominated, with liver (13.1%, 95% CrI: 4.9–45.5%), bowel (11.2%, 4.2–40.9%), spleen (11.1%, 4.1–40.4%), and kidney (8.7%, 3.2–34.3%) injuries most common; adrenal (3.4%) and pancreatic (1.4%) injuries were rare. Meta-regression showed higher injury probabilities with increasing age and male predominance. Injury Severity Score–based subgroup analyses yielded substantially lower IAI probabilities (11–12%), reflecting broader trauma populations. Certainty of evidence was moderate for overall IAI prevalence and low for other outcomes due to heterogeneity and sparse events.

**Conclusion:**

Although CT is highly sensitive for detecting IAIs in pediatric blunt abdominal trauma, low rates of intervention and mortality support selective CT use guided by validated decision rules and observation rather than routine imaging. Future research should prioritize multicenter prospective studies, pragmatic implementation of decision tools, and development of non-ionizing imaging alternatives to optimize CT use and minimize long-term risks in children.

**Supplementary Information:**

The online version contains supplementary material available at 10.1186/s13049-026-01578-5.

## Introduction

Trauma remains the leading cause of death in children beyond infancy, accounting for about 20,000 deaths annually in the United States. Additionally, approximately 500,000 hospital admissions each year [1]. Around 85% of pediatric trauma cases result from blunt mechanisms, with blunt abdominal trauma (BAT) representing a significant contributor to morbidity and mortality [2].

The assessment of acutely injured children presents unique challenges. Limited communication skills, heightened anxiety following the traumatic event, and decreased levels of consciousness may all compromise the reliability of physical examination.

Because missing intra-abdominal injuries (IAIs) can have severe consequences, computed tomography (CT) is frequently employed in the acute settings. CT is rapid, widely accessible, highly sensitive for detecting various types of pediatric Injuries, and is generally well interpreted by trauma surgeons. Nevertheless, it carries several limitations: high costs, increased utilization of hospital resources, and exposure to ionizing radiation. Radiation-related cancer risk is particularly concerning in younger patients, where long-term risk is amplified [3–5].

To address unnecessary radiation exposure, several clinical decision rules have been developed. These utilize variables such as mechanism of injury, clinical signs and symptoms, laboratory testing, and findings from the focused abdominal sonography for trauma (FAST) examination to support decision-making regarding CT of the abdomen and pelvis [6, 7]. However, unlike head trauma—where standardized decision tools such as the Pediatric Emergency Care Applied Research Network (PECARN) guidelines are widely implemented— no consensus approach exists for managing BAT in children [8].

In light of these uncertainties, there is a pressing need to synthesize the evidence on CT use in pediatric BAT. So, we conducted a systematic review and meta-analysis to estimate the prevalence of intra-abdominal injury in children with blunt trauma, and to evaluate the diagnostic yield, rates of missed injury, intervention requirements, and adverse outcomes associated with CT compared with alternative strategies.

## Methods

This systematic review and meta-analysis was conducted in accordance with the Preferred Reporting Items for Systematic Reviews and Meta-Analyses guidelines [9] and the Cochrane Handbook for Systematic Reviews [10]. The protocol was registered with PROSPERO (International Prospective Register of Systematic Reviews) under registration number CRD420251144940.

### Search strategy and literature search

A comprehensive literature search from inception to August 2025 including the following databases: PubMed, Web of Science, Cochrane Library and Scopus utilizing free-text keywords related to "Abdominal blunt trauma", "Computed tomography", and "pediatric". References were exported using EndNote 20 software (Clarivate, CA, USA) into a local library. Further more, manual searching was conducted to ensure that we included all relevant studies. The detailed search strategy is provided in Supplementary Table 1.

### Selection criteria and title and abstract screening

**Fig. 1:**
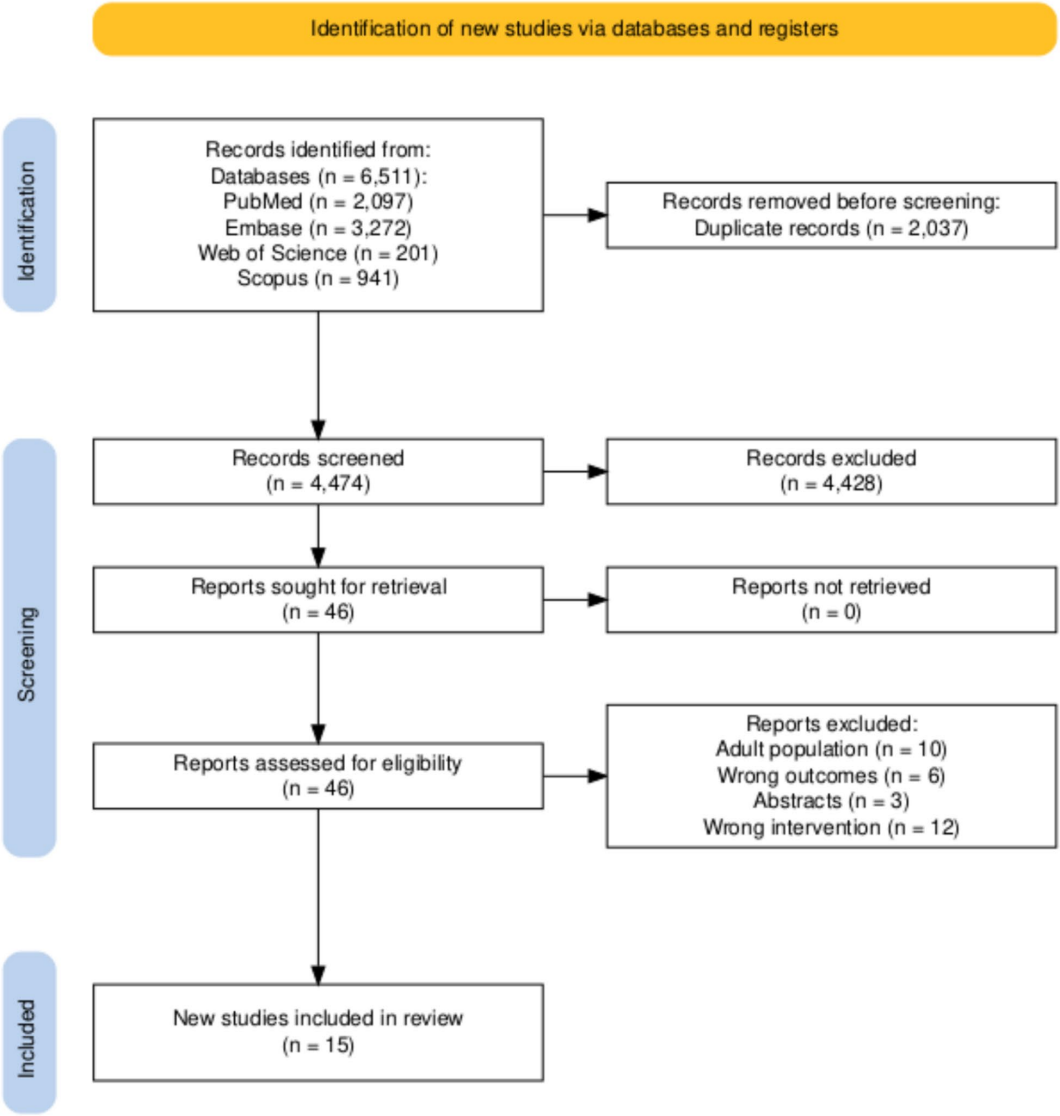
PRISMA flowchart of included studies

The screening step was performed using a Rayyan library. Any conflicts during the screening process were resolved through discussion among the two reviewers. When needed, input from a third reviewer was sought. Full-text articles were assessed to determine their eligibility for data extraction. The study selection process is depicted in a PRISMA flow diagram (Fig. 1). Eligible studies included pediatric patients aged 0–18 years with abdominal blunt trauma, excluding adults. Injury Severity Score (ISS) reporting was not required for study inclusion, as many pediatric blunt abdominal trauma studies focus on imaging utilization or diagnostic yield rather than overall trauma severity. Studies were included if they reported CT-based detection of intra-abdominal injury in pediatric blunt trauma populations, regardless of ISS availability. Studies were excluded if they focused on predominantly adult or neonatal populations, lacked original data (reviews, editorials, or letters), were case series, or failed to provide sufficient data to derive accuracy metrics.

### Data extraction

Two reviewers independently extracted data using a standardized form, including: Study details: (author, year, country, study design, setting, sample size). Patient characteristics: (age distribution, sex, mechanism of injury, abdominal blunt trauma prevalence). Intervention details: (CT abdomen and comparator used). Reported outcomes included abdominal tenderness, GCS, seatbelt sign, CT diagnostic yield for identifying IAI, rates of missed injury or need for intervention, CT utilization rate, length of stay and ED return.

### Risk of bias assessment

Methodological quality and risk of bias for included studies were independently assessed by ( MH& MA) using the QUADAS-2 tool to assess risk of bias in diagnostic accuracy studies [11].

### Data synthesis and statistical analysis

To estimate the pooled prevalence of intra-abdominal injury, Bayesian random-effects meta-analysis was used. This approach was chosen due to substantial heterogeneity across studies and the presence of sparse outcome data [12].

Given the substantial clinical and methodological heterogeneity across cohorts, a conventional fixed-effects approach would be inappropriate. Bayesian random-effects models are well suited for synthesizing heterogeneous prevalence data, as they explicitly model between-study variability rather than assuming a common underlying effect of total organ injuries per study as the event count and study sample size as the denominator. Weakly informative priors were used to prevent implausible estimates in sparse data settings, while remaining sufficiently broad so that results were driven primarily by the observed data. This approach is commonly recommended in Bayesian meta-analysis of small or heterogeneous datasets. A random intercept for each study captured study-specific deviations from the overall prevalence, This formulation models the probability of injury as a proportion, ensuring that estimates remain between 0 and 100%.

The model used a binomial likelihood with a logit link, where the number of intra-abdominal injuries was the event count and the study sample size served as the denominator. A random intercept accounted for study-specific variability. Weakly informative priors were specified to stabilize estimation without unduly influencing results.

Posterior distributions were estimated via Markov chain Monte Carlo (MCMC) sampling, with convergence assessed by trace plots and the Gelman–Rubin statistic (R̂ < 1.05). The posterior median and 95% credible intervals (CrIs) were reported as pooled effect estimates. Between-study heterogeneity was summarized on the logit scale. Posterior predictive checks were performed to assess model fit by comparing simulated outcomes with observed study-level data.

Where available, studies reporting ISS were included in a prespecified subgroup analysis stratified by low (≤ 8), moderate (9–15), and severe (> 15) ISS using a Bayesian multilevel logistic regression model to explore the association between injury severity and intra-abdominal injury prevalence. Detailed statistical analysis methods are presented in *Supplementary Methods*.

### Certainty of evidence

The certainty of evidence for key outcomes was assessed using the GRADE approach, considering risk of bias, indirectness, inconsistency, imprecision, and publication bias [13].

## Results

### Screening and study selection

A total of 6,511 studies were obtained following an initial search, of which 4,474 studies were screened following removal of duplicates. Forty-six studies were further evaluated using their full-texts of which 15 studies including 7,430 children were included for final qualitative and quantitative analysis [14–28].

### Baseline characteristics of included studies

The 15 included studies were published between 1993 and 2025 and were conducted across diverse geographical settings, predominantly in the United States, with single studies from South Africa, Turkey, and Canada. Study designs were mainly retrospective cohorts (*n* = 11), supplemented by prospective cohorts (*n* = 3) and a secondary analysis of a prospective cohort (*n* = 1). Sample sizes varied substantially, ranging from small single-center cohorts to large-scale datasets. The duration of follow-up also showed wide variability, spanning from as short as 1 month (Klein, 2025) to up to 11 years (Karmazyn, 2025; Lamoshi, 2024).

The mean age of participants ranged from infancy (1.2 years in Karmazyn, 2025) to adolescence (16.6 years in Kolousek, 2023). Male predominance was observed in most cohorts where reported, with proportions ranging between 37 and 70%. Injury Severity Scores (ISS), reported in a subset of studies, demonstrated heterogeneity, with mean values spanning from 6.5 (Edwards, 2021) to 17.1 (Kolousek, 2023). Detailed baseline characteristics are provided in Table 1.

**Table 1:** Summary and baseline characteristics of included studies

Study	Country	Study design	Sample size	Time of follow-up	Age (mean (SD))	Males (n(%))	ISS score (mean (SD))
Kolousek_2023	USA	Retrospective cohort	546	5 years	16.6 (1)	217 (67)	17.13 (11.68)
Ugalde_2025	USA	Secondary analysis of a prospective cohort	7,581	4 years	10.1 (4.8)	1446 (58)	NR
Edwards_2021	USA	Retrospective cohort	735	2 years	6.9	NR	6.5 (4.6)
Arnold_ 2013	South Africa	Retrospective cohort	117	8 years	NR	NR	NR
Karmazyn_2025	USA	Retrospective cohort	455	11 years	1.2 (0.99)	74 (59.67)	NR
Kuas_2022	Turkey	Prospective cohort	327	1 year	11 (5.96)	106 (37.45)	NR
Lamoshi_2024	USA	Retrospective cohort	726	11 years	12.65 (5.88)	494 (68)	12.93 (11.15)
Meyer_1993	USA	Prospective cohort	60	1 year	5.8	42 (70)	NR
Odia _2024	USA	Retrospective cohort	115	2 years	NR	NR	NR
Yancovich_2025	USA	Retrospective cohort	923	10 years	8.5	450 (66.4)	12.25 (10.31)
Beno_2022	Canada	Retrospective cohort	804	3 years	8.93 (6.4)	509 (63.3)	NR
McGrew_2015	USA	Retrospective cohort	1934	7 years	7.5 (4.8)	NR	NR
Holmes_2024	USA	Prospective cohort	7542	4 years	9.5 (6.23)	4290 (56.9)	NR
Otaibi_2025	USA	Retrospective cohort	483	5 years	13 (3)	281 (58.2)	9 (7.44)
Klein_2025	USA	Retrospective cohort	446	1 month	11.25 (4.42)	28 (65.12)	NR

### Quality assessment (QUADAS-2)

The quality assessment, performed using the QUADAS-2 tool, revealed varying levels of risk of bias and applicability concerns across the included studies. While the Index Test domain consistently showed a low risk of bias for all studies, concerns were noted in Participant Selection for Arnold et al. and Karmazyn et al. due to inappropriate selection criteria. The Reference Standard and Flow and Timing domains presented mixed results, with Ugalde et al. exhibiting unclear risks of bias and applicability concerns (Supplementary Fig. 1).

### Statistical modeling

**Fig. 2:**
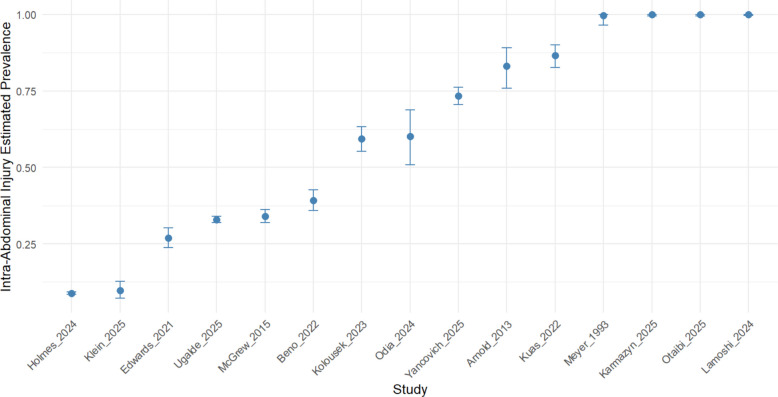
Study-specific posterior estimates of any intra-abdominal injury in pediatric blunt trauma. Points represent the posterior median prevalence of intra-abdominal injury in each study, derived from a Bayesian random-effects meta-analysis using a binomial likelihood with a logit link. Horizontal lines indicate the 95% credible intervals for each study, reflecting uncertainty in the estimate. Study IDs are abbreviated for clarity

The posterior median estimate of the pooled prevalence was 84.5%, with a 95% credible interval of 62% to 94%, indicating that the majority of pediatric patients sustaining blunt abdominal trauma experience at least one intra-abdominal injury. Study-specific deviations were notable, with high-risk cohorts such as Karmazyn et al. (2025) demonstrating a predicted prevalence of 99.8%, while lower-risk cohorts like Edwards et al. (2021) had a predicted prevalence of 6%. The between-study heterogeneity on the logit scale was estimated at 14.2, reflecting the variability in patient populations, injury severity, and study design across the included datasets. Posterior predictive checks confirmed that the model accurately reproduced the distribution of observed injuries, with a mean posterior predictive total of 622 injuries, supporting the model’s validity for predicting outcomes in future pediatric populations (Supplementary Table 2) (Fig. 2).

### Organ-specific injury prevalence

To describe patterns of abdominal organ injury following pediatric blunt trauma, we estimated the prevalence of injuries to the liver, spleen, bowel, kidney, adrenal glands, and pancreas across the included studies. For each study, the number of injuries in a given organ was evaluated relative to the total number of patients in that study. Differences between studies—such as variation in patient characteristics, injury severity, and clinical practice—were accounted for by allowing each study to have its own baseline risk.

Model estimates were converted to probabilities (percentages) to support clinical interpretation and are reported as median values with 95% credible intervals (CrIs), which reflect the range of values compatible with the observed data.

**Fig. 3:**
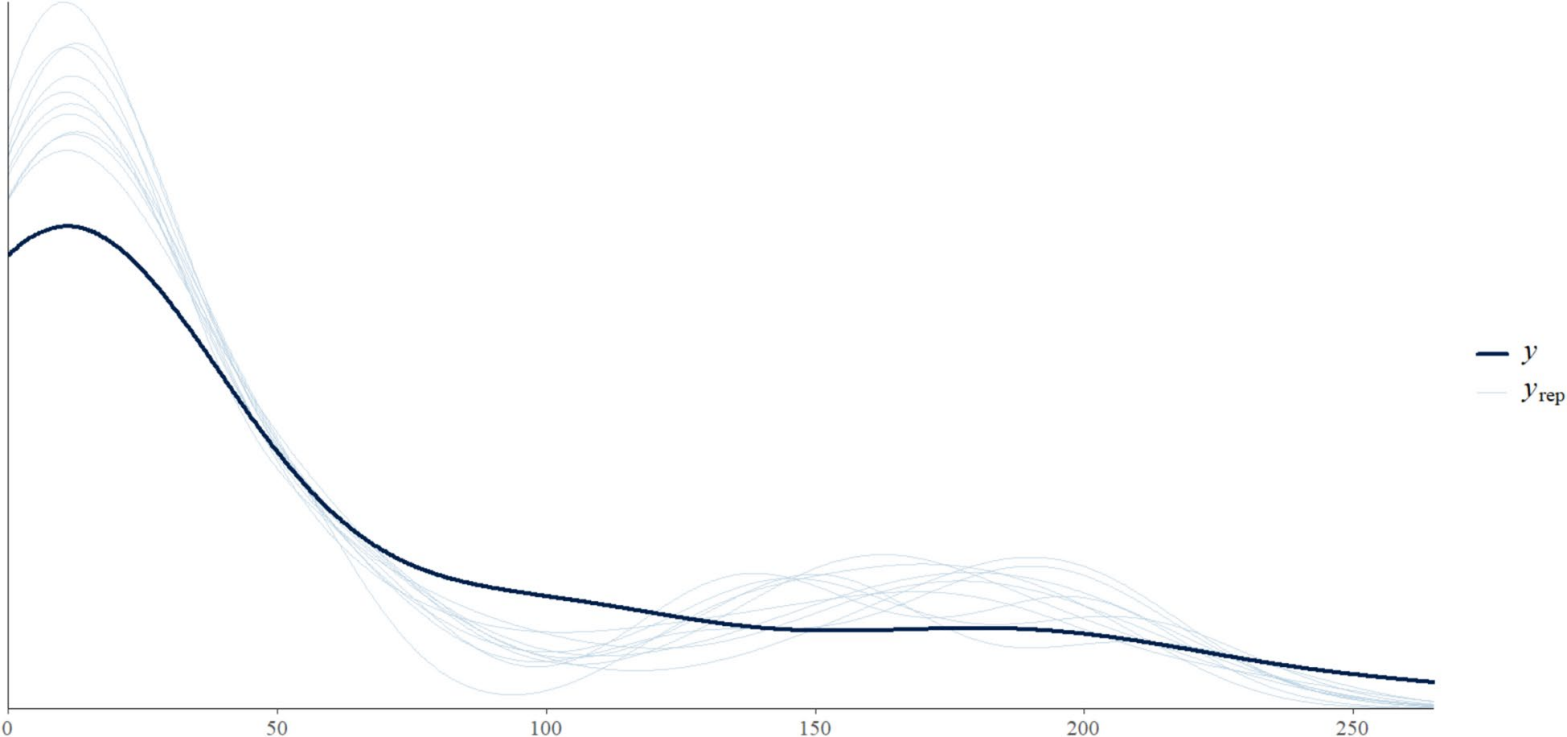
Posterior predictive check for the organ-specific injury model. The dark line represents the observed distribution of injury counts, while light blue lines show replicated data simulated from the posterior. The close alignment supports the adequacy of the Bayesian model

Results are summarized in Table 2. Solid organ injuries were most frequently observed. Liver injuries had the highest estimated prevalence (13.1%, 95% CrI 4.9–45.5%), followed by bowel (11.2%, 95% CrI 4.2–40.9%), spleen (11.1%, 95% CrI 4.1–40.4%), and kidney injuries (8.7%, 95% CrI 3.2–34.3%). Injuries to the adrenal glands and pancreas were uncommon, with estimated prevalences of 3.4% (95% CrI 1.2–16.2%) and 1.4% (95% CrI 0.5–7.4%), respectively (Fig. 3). Comparison of observed and model-predicted injury counts showed good agreement, indicating that the model adequately captured organ-specific injury patterns across the diverse study populations.

**Table 2:** Posterior prevalence estimates of organ-specific injuries (back-transformed probabilities)

Organ	Median	95% CrI (lower–upper)
Adrenal	0.034	0.012 – 0.162
Bowel	0.112	0.042 – 0.409
Kidney	0.087	0.032 – 0.343
Liver	0.131	0.049 – 0.455
Pancreas	0.014	0.005 – 0.074
Spleen	0.111	0.041 – 0.404

Median posterior prevalence estimates were:

- Liver: 12.7% (95% CrI 4.7–42.4%)
- Spleen: 10.6% (3.9–37.8%)
- Bowel: 10.7% (4.0–37.9%)
- Kidney: 8.3% (3.0–31.5%)
- Adrenal: 3.3% (1.1–14.3%)
- Pancreas: 1.3% (0.4–6.5%)

To contextualize the clinical relevance of these estimates, we calculated the probability that each organ’s prevalence exceeds a 10% threshold. Liver injuries had a 65% chance, bowel 56%, and spleen 55%, while kidney, adrenal, and pancreas were less likely to exceed this threshold (38%, 7%, and 1%, respectively) (Supplementary Table 3). This approach quantifies the likelihood of clinically meaningful injury, supporting evidence-based decisions in pediatric trauma care.

### Model stability and convergence

We evaluated whether the statistical model produced stable and reliable estimates across repeated simulations. Standard diagnostic checks showed that all model parameters converged well and that results were consistent across independent runs. Measures of convergence indicated minimal variation between simulations, and there was no evidence of numerical instability during model fitting.

To further test robustness, the model was re-estimated using more conservative computational settings. These sensitivity analyses produced results that were essentially unchanged, supporting the reliability of the estimated injury probabilities (Supplementary Table 4).

### Model robustness and influence of individual studies

To assess how well the model predicts new data and whether any single study unduly influenced the results, we performed leave-one-out cross-validation. This approach evaluates model performance by repeatedly refitting the model while omitting one observation at a time.

**Fig. 4:**
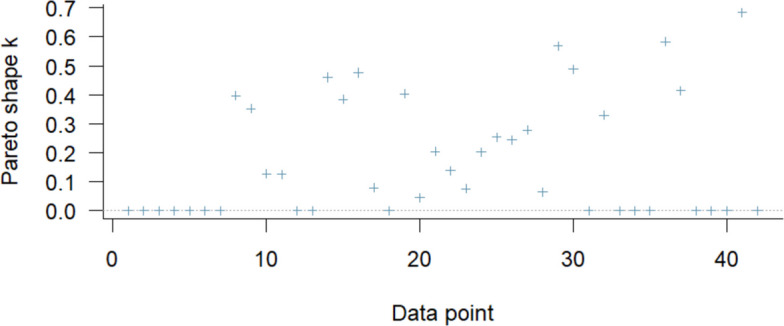
Leave-One-Out (LOO) cross-validation for the organ-specific injury model. The LOO plot visualizes the Pareto k diagnostics for each observation, assessing their influence on model predictions. Most observations fall within the “good” range (k < 0.5), with a few classified as “ok” (0.5 ≤ k < 0.7), indicating minor influence. No observations were “bad” or “very bad,” suggesting the model’s posterior estimates are robust and not overly dependent on any single study. This supports the reliability of predicted organ-specific injury probabilities in pediatric blunt trauma

The analysis indicated that nearly all observations contributed appropriately to the overall model fit, with no studies exerting excessive influence on the results. Overall predictive performance was stable, and the conclusions were not driven by any individual study (Fig. 4). These findings support the robustness of the organ-specific injury estimates across heterogeneous study populations.

### Intervention and mortality models

We next examined the prevalence of interventions and mortality in pediatric blunt trauma by examining intervention use and mortality using study-level models that account for differences between studies. Study-level random intercepts accounted for heterogeneity across studies, and weakly informative priors stabilized estimation given the small number of included studies.

For interventions, data from 6 studies were analyzed. The posterior median probability of receiving an intervention was 7.7% (back-transformed from the logit intercept of − 2.49), with study-level heterogeneity (SD on the logit scale = 0.97) reflecting variation in practice patterns and patient severity across cohorts.

**Fig. 5:**
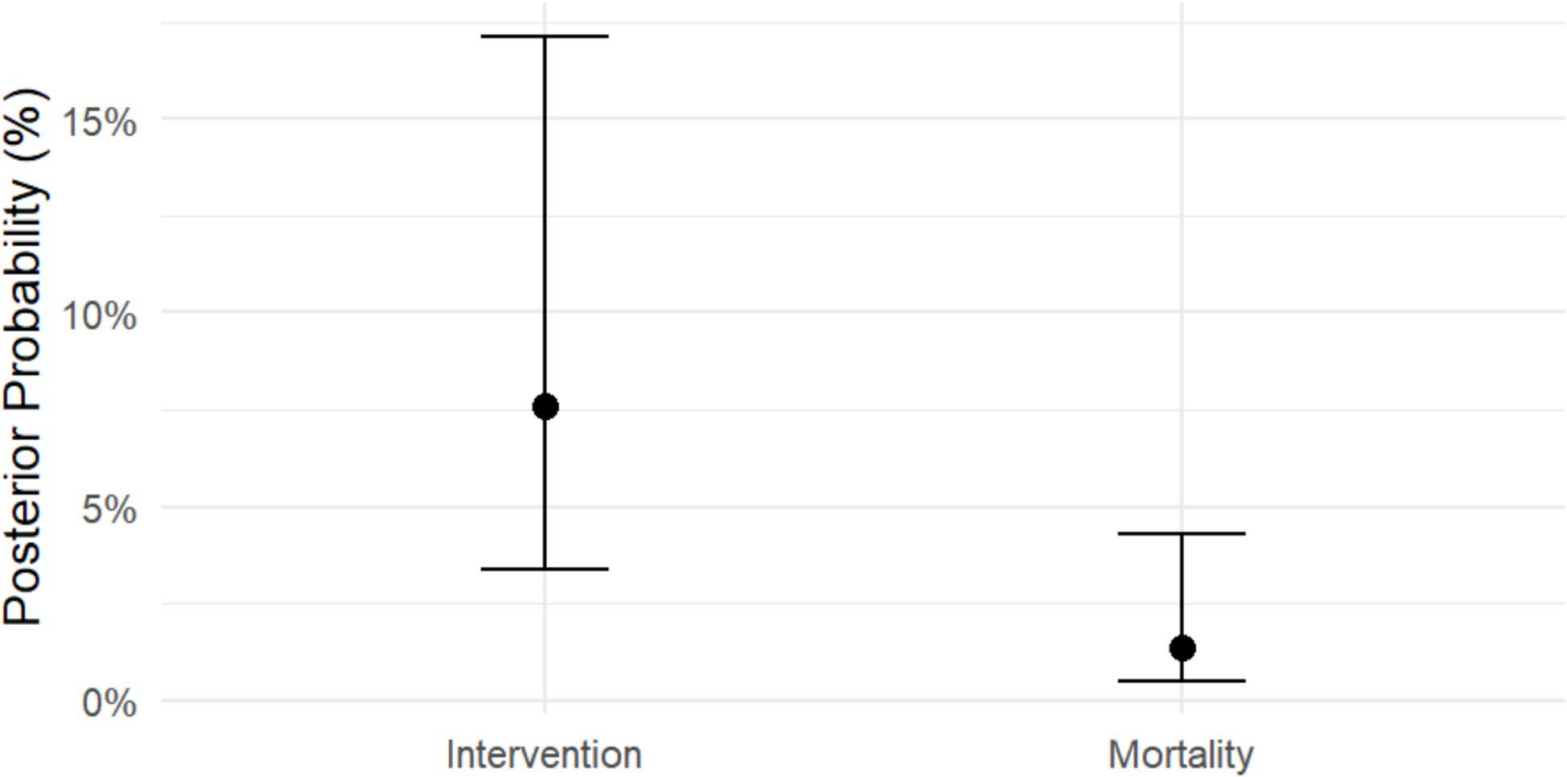
Posterior probabilities of intervention and mortality in pediatric blunt trauma. Points represent the posterior median probability of receiving an intervention or experiencing mortality, back transformed from the logit scale. Error bars indicate 95% credible intervals, reflecting uncertainty in each estimate. The plot allows direct visual comparison of the expected likelihood of intervention versus mortality across pediatric blunt trauma populations

For mortality, data from 8 studies were included. The posterior median probability of death was 1.4% (logit intercept = − 4.25), with study-level heterogeneity of 1.45 on the logit scale, indicating variability in case-mix and trauma severity. Posterior predictive checks confirmed that both models adequately reproduced the observed study-level counts, supporting the use of these estimates as descriptive benchmarks for clinical expectations in pediatric blunt trauma. (Supplementary Table 5, Fig. 5).

### Bayesian meta-regression of age and male proportion

To explore whether study-level patient characteristics influenced the probability of sustaining any intra-abdominal injury, we fitted a Bayesian meta-regression including mean age and proportion of male patients as predictors. The model included a random intercept for each study to account for between-study variability. Posterior draws were back-transformed from the logit to the probability scale to facilitate clinical interpretation.

**Fig. 6:**
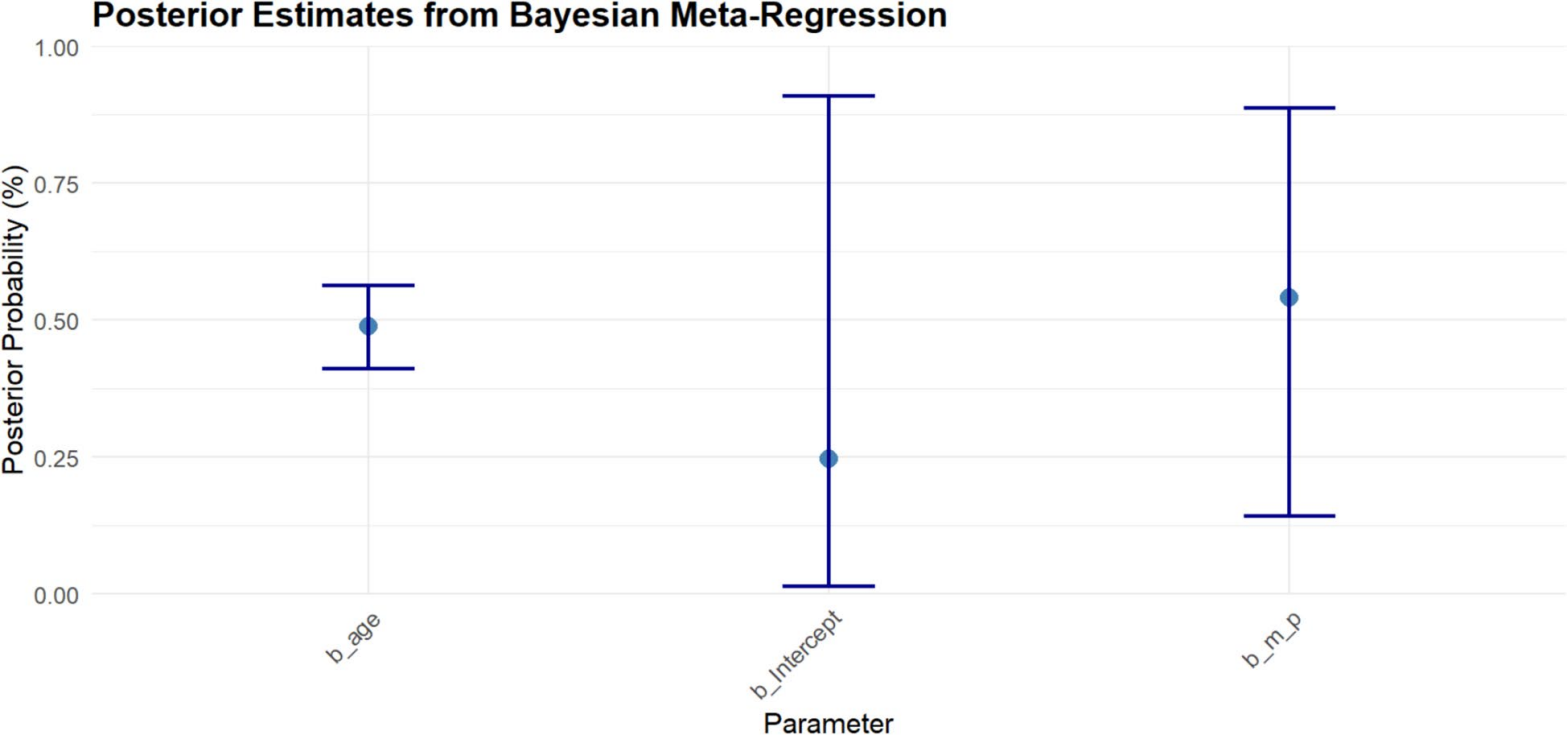
Posterior estimates from Bayesian meta-regression of intra-abdominal injuries in pediatric blunt trauma. Points represent the posterior median probability for each parameter (intercept, age, and male proportion), with vertical lines indicating the 95% credible intervals. Probabilities are presented on a percentage scale, providing an interpretable measure of the expected effect of each covariate on injury prevalence while accounting for between-study variability

The posterior median probability of any intra-abdominal injury for the baseline (intercept) was 24.8% (95% CrI 1.5–90.9%). Age was associated with a higher probability of injury, with a median posterior estimate of 48.8% (95% CrI 41.2–56.2%), while studies with a higher proportion of male patients had a median posterior probability of 54.0% (95% CrI 14.3–88.7%) (Supplementary Table 6, Fig. 6). These associations reflect study-level relationships and should not be interpreted as individual-level risk factors.

### Subgroup analysis: probability of injury by ISS

We conducted a subgroup analysis to examine the association between Injury Severity Score (ISS) and the likelihood of sustaining any intra-abdominal injury in pediatric blunt trauma. ISS was categorized as Low (ISS ≤ 8), Moderate (ISS 9–15), and Severe (ISS > 15). Using a Bayesian multilevel logistic regression model, the number of injured patients per study was modeled as a function of ISS group, with study-level random intercepts to account for between-study variability. Posterior estimates were back-transformed from the logit scale to probabilities to facilitate clinical interpretation. Model diagnostics confirm convergence with model checks indicated stable and reliable estimation across ISS groups, supporting the reliability of the estimates.

The estimated probability of sustaining an intra-abdominal injury was 11% (95% credible interval [CrI] 2–35%) for the Low ISS group, 12% (95% CrI 1–49%) for the Moderate ISS group, and 12% (95% CrI 1–54%) for the Severe ISS group. Although probabilities were slightly higher in the Moderate and Severe groups compared to the Low group, the overlapping credible intervals indicate that these differences were not statistically significant. This suggests that, within the limits of the available data, ISS alone may not be a strong predictor of intra-abdominal injury once study-level heterogeneity is considered.

### Publication bias assessment

Visual inspection suggested marked asymmetry in prevalence estimates, consistent with substantial heterogeneity and small-study effects rather than classical publication bias. Quantitative assessment confirmed substantial heterogeneity across studies, reflecting marked variability in study populations, injury severity, and reporting (Supplementary Fig. 2).

### GRADE assessment of evidence

We assessed the certainty of evidence for all outcomes using the GRADE framework, considering risk of bias, inconsistency, indirectness, imprecision, and publication bias. For the overall prevalence of intra-abdominal injury, the evidence was moderate, reflecting high heterogeneity across studies and some asymmetry in study reporting, although modeling approaches accounted for heterogeneity and sparse data.

For organ-specific injuries, the certainty of evidence was generally low, primarily due to wide credible intervals, sparse events, and heterogeneity among studies. Solid organ injuries—particularly liver, spleen, and bowel—showed moderate posterior probabilities (> 10%) for clinically meaningful prevalence, whereas adrenal and pancreatic injuries were rare, limiting the robustness of these estimates.

Intervention and mortality outcomes were also rated as low-certainty, given the small number of studies, rare events, and wide posterior credible intervals, despite stable model convergence and adequate posterior predictive checks.

Bayesian meta-regression analyses examining the influence of age and male proportion were rated as low-certainty. While both older age and higher proportion of male patients were associated with increased probability of intra-abdominal injury, credible intervals were wide, reflecting the limited number of included studies and inherent uncertainty in study-level covariate effects (Table 3).

**Table 3:** Grade assessment table

Outcome	Pooled Estimate (95% CrI / CI)	Studies (n)	Certainty of Evidence (GRADE)	Key Considerations
Any intra-abdominal injury	84.5% (62–94%)	15	Moderate ⬤⬤⬤◯	High heterogeneity; potential publication bias; Bayesian approach mitigates sparse data
Liver injury	12.7% (4.7–42.4%)	15	Low ⬤⬤◯◯	Solid organ; moderate uncertainty; posterior probability > 10% = 65%
Spleen injury	10.6% (3.9–37.8%)	15	Low ⬤⬤◯◯	Posterior probability > 10% = 55%; heterogeneity across cohorts
Bowel injury	10.7% (4.0–37.9%)	15	Low ⬤⬤◯◯	Sparse events; moderate credible interval width
Kidney injury	8.3% (3.0–31.5%)	15	Low ⬤⬤◯◯	Posterior probability > 10% = 38%; lower prevalence
Adrenal injury	3.3% (1.1–14.3%)	15	Low ⬤⬤◯◯	Rare outcome; limited data; wide CrI
Pancreas injury	1.3% (0.4–6.5%)	15	Low ⬤⬤◯◯	Very rare; posterior probability > 10% = 1%
Interventions	7.7% (3.4–16.4%)	6	Low ⬤⬤◯◯	Small study number; heterogeneity present; stable posterior estimates
Mortality	1.4% (0.5–3.9%)	8	Low ⬤⬤◯◯	Rare; credible intervals wide; robust posterior predictive checks
Age (meta-regression)	48.8% (41.2–56.2%)	15	Low ⬤⬤◯◯	Suggests increased prevalence with older age; moderate uncertainty
Male proportion (meta-regression)	54.0% (14.3–88.7%)	15	Low ⬤⬤◯◯	Higher prevalence with more male patients; wide credible interval
Probability of injury by ISS subgroup	Low: 11% (2–35%); Moderate: 12% (1–49%); Severe: 12% (1–54%)	12	Low ⬤⬤◯◯	Bayesian multilevel model with study-level random intercepts; overlapping credible intervals indicate no significant difference between ISS groups; highlights variability and uncertainty in predicting injury by ISS alone

## Discussion

In this systematic review and Bayesian meta-analysis we synthesized data from 15 studies (7,430 children) to describe the epidemiology, organ distribution, and outcomes of intra-abdominal injury (IAI) after pediatric blunt abdominal trauma (BAT), and to examine the downstream consequences of computed tomography (CT)–based evaluation. Our modelling noted a higher proportion of solid-organ injuries (liver, spleen, bowel > kidney, adrenal, pancreas). Further, the proportion of children receiving an intervention or dying is low relative to the number of injuries detected on imaging, suggesting promising outcomes. However, there was a large between-study heterogeneity in reported prevalence estimates and CT use. These findings reinforce the diagnostic power of CT while simultaneously highlighting persistent uncertainty about who benefits from immediate CT versus observation/targeted imaging, and the potential for substantial practice variation and harms from overuse.

Our pooled posterior median for any intra-abdominal injury was high at 84.5%. Studies report varying prevalences; certain studies report a low prevalence at 2.99% [29], while others noted a prevalence up to 12 to 16% [30]. Several contemporary multicentre and registry studies report that the proportion of children with IAI among all ED presentations for blunt torso trauma is usually in the range of 6 to 12% [31]. The higher prevalence noted in our analysis could be due to the underlying studies selected. Many included cohorts were selected subsets, for example, children who underwent CT or were evaluated at tertiary trauma centres, or retrospective single-centre series of high-risk patients [32]. Differences in the case definition of intra-abdominal injury (any radiographic abnormality versus only clinically important injuries requiring intervention) also create large downstream differences in pooled estimates [33]. These sources of variation and selection are well described in the recent literature and explain why pooled prevalences for IAI vary widely between reviews depending on the denominator and outcome definition. [34]. A key point in interpreting our findings is the apparent discrepancy between the overall pooled prevalence of intra-abdominal injury (84.5%) and the much lower estimates (11–12%) observed in subgroup analyses stratified by injury severity. This difference reflects variation in denominators across studies. The pooled estimate was derived from cohorts in which nearly all children were either imaged with CT or triaged as high-risk at tertiary trauma centres, inherently inflating the prevalence. By contrast, the subgroup estimates reflect broader denominators, closer to all blunt trauma presentations, and therefore better approximate the population-level burden. This pattern aligns with prior multicentre cohorts such as PECARN, where the prevalence of intra-abdominal injury among all ED blunt trauma presentations was consistently in the single digits to low teens [26]. Thus, our pooled estimates should be interpreted not as a baseline risk for every child presenting with blunt abdominal trauma, but rather as the prevalence conditional on being imaged or identified as high-risk.

Our analyses found that solid-organ injuries (liver, spleen) and hollow-viscus/mesenteric injuries are the common radiologic findings after BAT, with pancreatic and adrenal injuries comparatively rare. This organ distribution mirrors contemporary single-institution and multicentre trauma reports and imaging reviews that emphasize the predominance of hepatic and splenic injuries in blunt pediatric abdominal trauma [35, 36]. Liver and splenic trauma are often managed by a non-operative approach, proving to be successful in nearly 98% of all children. On the other hand, some organ injuries (small bowel or mesenteric) may be radiographically subtle and escape detection on initial FAST/CT or present later, reinforcing the need for careful observation when clinical suspicion persists [37, 38].

We estimated a posterior median probability of intervention of 7.7% and mortality 1.4% across the datasets. These values align with contemporary pediatric trauma literature showing that the overwhelming majority of solid-organ injuries in children are managed non-operatively and that operative intervention is uncommon in center-level series [39]. Mortality attributable to isolated blunt abdominal injury in modern pediatric trauma systems is low; deaths are primarily seen in the highest-severity polytrauma cases [29]. Our results reiterate that CT findings alone should not be equated with the need for surgery; clinical status and physiology remain paramount in deciding operative care [40].

Our results reinforce the status of contrast-enhanced abdominal CT as the most sensitive imaging modality for identifying a broad range of IAIs in hemodynamically stable children; modern CT protocols optimized for pediatrics remain the reference standard for anatomic diagnosis and grading of intra-abdominal injuries. At the same time, alternative approaches like clinical risk-stratification, focused abdominal sonography for trauma, and laboratory testing can safely reduce CT utilization when applied to appropriately selected patients [41]. Recent reviews show substantial variation in CT use (often > 30–40% of pediatric blunt trauma patients in many centres), and efforts to rationalize imaging remain a priority [42].

The randomized trial by Holmes et al. found that routine use of FAST in the initial evaluation of stable pediatric blunt-torso trauma did not significantly change the clinical outcomes, largely because FAST sensitivity for many pediatric injuries is modest and clinicians frequently pursue CT despite negative sonography [43]. Systematic reviews since then report moderate sensitivity for FAST in children and emphasize operator dependence and the test’s better performance for hemoperitoneum than for isolated parenchymal or hollow-viscus injuries [44].

The extreme between-study heterogeneity we observed reflects clinically meaningful differences across studies in case-mix (ED vs trauma centre vs registry), age distributions, mechanism of injury, thresholds for CT and imaging protocols (for example: with/without oral contrast, arterial vs portal venous phases). Recent work documents large disparities in CT utilization across hospital types and regions, which further amplifies heterogeneity in reported prevalence and performance metrics [34].

Key strengths include the use of Bayesian hierarchical models that are well-suited for sparse and heterogeneous event data. The Bayesian framework allowed direct probability statements (posterior medians and credible intervals), incorporation of weakly informative priors to stabilize estimation in small samples, and rich model checking to assess fit and influence. Despite these strengths, several important limitations constrain interpretation. First, the primary studies we pooled were heterogeneous in design (retrospective vs prospective) and imaging protocols, which was reflected in our large between-study variance and downgraded GRADE assessments for many outcomes. Second, selection bias is probable because many source cohorts included only children who underwent CT (or were treated at tertiary centres). Third, a handful of small studies with extreme prevalences likely exaggerated pooled estimates; small-study effects and potential publication bias are plausible. Fourth, some outcomes (organ-specific prevalence, interventions, mortality) were reported in relatively few studies, limiting precision and increasing reliance on model regularization. Fifth, long-term harms from CT, like radiation-related effects cannot be estimated from observational trauma cohorts and require linkage or modeling studies with very large denominators; our work cannot directly quantify those long-term risks. ISS was not reported in a substantial proportion of included studies, reflecting a limitation of the pediatric trauma imaging literature. Excluding these studies would have introduced selection bias by disproportionately retaining registry-based or high-acuity trauma center cohorts, thereby reducing generalizability. Although ISS-based subgroup analyses were performed where data permitted, residual confounding by injury severity cannot be fully excluded. Finally, variable reporting hampered our ability to perform patient-level meta-analysis, subgrouping, or robust adjustment for confounders (like ISS, mechanism, hemodynamic status).

CT remains indispensable for detecting a broad spectrum of IAIs in children, but routine, indiscriminate use exposes children to possibly harmful radiation and downstream cascades without commensurate benefit for most low-risk patients. Implementation of validated clinical decision instruments (PECARN blunt-torso rule) [26], adherence to pediatric-optimized CT protocols (dose-reduction and phase tailoring), judicious use of adjuncts (FAST, laboratory testing, serial abdominal exams), and shared decision-making with families should reduce low-value imaging while maintaining patient safety. Prospective multicentre implementation and impact studies are needed to quantify how rule adoption changes clinical outcomes, CT utilization, missed-injury rates, and resource use in diverse settings.

The potential role of emerging, non-ionizing and portable diagnostic technologies in the early detection of severe abdominal injury was increasingly recognized in recent literature. Advances in POCUS had extended beyond conventional FAST, with systematic reviews demonstrating the feasibility of automated and AI-assisted ultrasound algorithms for detecting intra-abdominal hemorrhage, potentially improving sensitivity and operator independence in prehospital and emergency settings [45]. In parallel, multivariate non-invasive physiologic monitoring technologies, including bioimpedance-based approaches, were shown in experimental models to detect occult hemorrhage before overt clinical deterioration, suggesting a role in early triage and decision-making [46]. Experimental studies also demonstrated that microbubble contrast-enhanced ultrasound could substantially improve the detection of active hemorrhage compared with conventional sonography, highlighting a possible future adjunct to bedside imaging [47]. Beyond ultrasound, microwave-based wearable systems were shown in animal models to detect and monitor traumatic abdominal injuries continuously, offering a novel, radiation-free approach for field or transport monitoring [48]. Additionally, advances in portable CT technology were increasingly reported, with modern scanners enabling bedside or near-patient cross-sectional imaging in critical care, trauma, and austere environments, potentially narrowing the gap between prehospital assessment and definitive imaging [49]. Although most of these technologies remained investigational or in early clinical adoption, their maturation was anticipated to meaningfully influence future pediatric trauma workflows by complementing or selectively reducing reliance on conventional hospital-based CT.

There is a need for large, prospective multicentre datasets that collect standardised, patient-level definitions for imaging, clinical findings, ISS, and outcomes so reliable predictive models can be built and compared. Further, randomized or pragmatic implementation trials that evaluate decision-rule deployment and its effect on CT rates and missed-injury outcomes in routine care can strengthen current evidence. Long-term epidemiologic linkage studies can aid in quantifying lifetime cancer risk from pediatric CT in modern low-dose practice.

## Conclusion

In this meta-analysis of 7,430 children across 15 studies, the pooled prevalence of intra-abdominal injury was 84.5%, with solid-organ injuries (liver, spleen) predominating. Despite this, the probability of acute intervention was only 7.7%, and mortality was 1.4%, underscoring the gap between radiographic detection and clinical significance. These findings support selective CT guided by validated rules and observation strategies rather than routine imaging. Future work should prioritize prospective multicentre data, pragmatic implementation of decision tools, and development of safe non-ionizing imaging alternatives while optimizing pediatric CT dose and monitoring long-term risks.

## Supplementary Information

Supplementary Material 1.

Supplementary Material 2.

## Data Availability

All data generated or analyzed during this study are included in this published article and its supplementary information files. Additional details are available from the corresponding author on reasonable request.

## Abbreviations

BAT: Blunt Abdominal Trauma
CrI: Credible Interval
CT: Computed Tomography
ED: Emergency Department
FAST: Focused Assessment with Sonography for Trauma
GRADE: Grading of Recommendations, Assessment, Development, and Evaluations
IAI: Intra-abdominal Injury
ISS: Injury Severity Score
LOO: Leave-One-Out cross-validation
