# Supplementary material for: Computed tomography in pediatric blunt abdominal trauma: current evidence, challenges, and future directions — a systematic review and meta-analysis
Source: Scand J Trauma Resusc Emerg Med. 2026 Feb 7;34:61. doi: 10.1186/s13049-026-01578-5 (PMC13011297; doi:10.1186/s13049-026-01578-5)
Supplement: Supplementary file 1 — Supplementary Material 1. [file 13049_2026_1578_MOESM1_ESM.docx]

**Supplementary Methods**

**Modeling Framework**

All analyses were conducted using Bayesian hierarchical models. This framework was chosen because it is well suited for heterogeneous observational data, allows explicit modeling of between-study variability, and provides coherent uncertainty quantification. Rather than assuming a single common prevalence across studies, the models allow true outcome probabilities to vary while estimating an overall distribution.

**Outcome Modeling and Link Function**

Outcomes were modeled as proportions, defined by the number of observed events relative to the total number of patients within each study. A binomial data model was used, which is appropriate for count-based outcomes and ensures that predicted values remain within valid probability bounds.

Probabilities were modeled on a transformed scale using a logit link. This transformation allows probabilities to be modeled on an unrestricted scale while ensuring that all back-transformed estimates lie between 0% and 100%. Results were converted back to probabilities to facilitate clinical interpretation.

**Hierarchical Structure and Heterogeneity**

Between-study heterogeneity was addressed using random effects, allowing each study to have its own baseline risk. This hierarchical structure enables partial pooling, whereby estimates from smaller or less precise studies are stabilized without overwhelming information from larger studies.

**Organ-Specific Models**

Organ-specific injury prevalence was estimated within a single hierarchical framework that included organ type as a fixed effect and study as a random effect. This allowed simultaneous estimation across organs while accounting for correlation within studies and borrowing strength across organ categories.

**Meta-Regression and Subgroup Analyses**

Exploratory meta-regression analyses were conducted to examine whether study-level characteristics, such as mean age or proportion of male patients, were associated with variation in injury prevalence. These analyses reflect ecological, study-level associations and are intended to be hypothesis-generating rather than causal.

Subgroup analyses based on Injury Severity Score (ISS) categories were performed using multilevel models that estimated separate baseline risks for each subgroup while retaining partial pooling across studies.

**Prior Specification**

Weakly informative priors were used to regularize estimation and improve numerical stability, particularly for rare outcomes and small numbers of studies. These priors constrain parameters to clinically plausible ranges while allowing the observed data to dominate inference when sufficient information is available.

**Computational Estimation (HMC)**

Model estimation was performed using Hamiltonian Monte Carlo (HMC), a gradient-based sampling algorithm designed for efficient exploration of complex posterior distributions. HMC is particularly well suited for hierarchical models and provides stable estimation with reduced autocorrelation compared with simpler sampling approaches.

**Model Checking and Robustness**

Model reliability was evaluated using standard convergence diagnostics and posterior predictive checks to ensure stable estimation and adequate fit to the observed data. Predictive robustness was further assessed using leave-one-out cross-validation to identify potentially influential studies.
