# Supplementary material for: Computed tomography in pediatric blunt abdominal trauma: current evidence, challenges, and future directions — a systematic review and meta-analysis
Source: Scand J Trauma Resusc Emerg Med. 2026 Feb 7;34:61. doi: 10.1186/s13049-026-01578-5 (PMC13011297; doi:10.1186/s13049-026-01578-5)

**Supplementary files**

**Supplementary table 1:** Search strategy for each database

| **Database** | **Advanced Search Strategy** |
| --- | --- |
| **PubMed** | ("pediatric*" OR "paediatric*" OR "child*" OR "neonat*" OR "infant*" OR "toddler*" OR "preschool" OR "pre-school" OR "juvenile" OR "young adult*") AND ("tomography, x-ray computed"[MeSH Terms] OR "CT scan"[Title/Abstract] OR "CT-scan"[Title/Abstract] OR "computed tomography"[Title/Abstract] OR "computerized tomography"[Title/Abstract]) AND ("trauma center*"[Title/Abstract] OR "pediatric trauma center*"[Title/Abstract] OR "adult trauma center*"[Title/Abstract] OR "trauma unit*"[Title/Abstract] OR "pediatric trauma unit"[Title/Abstract] OR "adult trauma unit*"[Title/Abstract] OR "ED"[Title/Abstract] OR "emergency department"[Title/Abstract] OR "accident and emergency"[Title/Abstract] OR "A&E"[Title/Abstract] OR "emergency room"[Title/Abstract]) AND ("wounds and injuries"[MeSH Terms] OR "wounds, nonpenetrating"[MeSH Terms] OR "blunt trauma"[Title/Abstract] OR "blunt injury"[Title/Abstract] OR "nonpenetrating trauma"[Title/Abstract] OR "non-penetrating trauma"[Title/Abstract] OR "nonpenetrating injury"[Title/Abstract] OR "non-penetrating injury"[Title/Abstract]) |
| **Scopus** | (TITLE-ABS-KEY(pediatric* OR paediatric* OR child* OR neonat* OR infant* OR toddler* OR preschool OR pre-school OR juvenile OR "young adult*") AND TITLE-ABS-KEY("tomography, x-ray computed" OR "CT scan" OR "CT-scan" OR "computed tomography" OR "computerized tomography") AND TITLE-ABS-KEY("trauma center*" OR "pediatric trauma center*" OR "adult trauma center*" OR "trauma unit*" OR "pediatric trauma unit" OR "adult trauma unit*" OR "ED" OR "emergency department" OR "accident and emergency" OR "A&E" OR "emergency room") AND TITLE-ABS-KEY("wounds and injuries" OR "wounds, nonpenetrating" OR "blunt trauma" OR "blunt injury" OR "nonpenetrating trauma" OR "non-penetrating trauma" OR "nonpenetrating injury" OR "non-penetrating injury")) |
| **Web of Science (WoS)** | TS=(pediatric* OR paediatric* OR child* OR neonat* OR infant* OR toddler* OR preschool OR pre-school OR juvenile OR "young adult*") AND TS=("tomography, x-ray computed" OR "CT scan" OR "CT-scan" OR "computed tomography" OR "computerized tomography") AND TS=("trauma center*" OR "pediatric trauma center*" OR "adult trauma center*" OR "trauma unit*" OR "pediatric trauma unit" OR "adult trauma unit*" OR "ED" OR "emergency department" OR "accident and emergency" OR "A&E" OR "emergency room") AND TS=("wounds and injuries" OR "wounds, nonpenetrating" OR "blunt trauma" OR "blunt injury" OR "nonpenetrating trauma" OR "non-penetrating trauma" OR "nonpenetrating injury" OR "non-penetrating injury") |
| **Embase (Ovid)** | (('pediatric*' OR 'paediatric*' OR 'child*' OR 'neonat*' OR 'infant*' OR 'toddler*' OR 'preschool' OR 'pre-school' OR 'juvenile' OR 'young adult*') AND ('tomography, x-ray computed'/exp OR 'CT scan':ab,ti OR 'CT-scan':ab,ti OR 'computed tomography':ab,ti OR 'computerized tomography':ab,ti) AND ('trauma center*':ab,ti OR 'pediatric trauma center*':ab,ti OR 'adult trauma center*':ab,ti OR 'trauma unit*':ab,ti OR 'pediatric trauma unit':ab,ti OR 'adult trauma unit*':ab,ti OR 'ED':ab,ti OR 'emergency department':ab,ti OR 'accident and emergency':ab,ti OR 'A&E':ab,ti OR 'emergency room':ab,ti) AND ('wounds and injuries'/exp OR 'wounds, nonpenetrating'/exp OR 'blunt trauma':ab,ti OR 'blunt injury':ab,ti OR 'nonpenetrating trauma':ab,ti OR 'non-penetrating trauma':ab,ti OR 'nonpenetrating injury':ab,ti OR 'non-penetrating injury':ab,ti)) |

Supplementary Table 2. Bayesian Random-Effects Model Estimates for Any Intra-Abdominal Injury in Pediatric Blunt Trauma

| **Parameter** | **Logit Mean (SD)** | **Back-transformed Median Prevalence (%)** | **95% Credible Interval (%)** |
| --- | --- | --- | --- |
| Overall intercept (pooled) | 1.7 (0.9) | 84.5 | 62 – 94 |
| Between-study SD (logit scale) | 14.2 | – | – |
| Example study: Karmazyn_2025 | 6.2 | 99.8 | – |
| Example study: Edwards_2021 | -2.7 | 6.0 | – |

Supplementary Table 3. Posterior probability of clinically meaningful injury (>10%)

| **Organ** | **Probability >10%** |
| --- | --- |
| Liver | 65.4% |
| Spleen | 54.5% |
| Bowel | 55.5% |
| Kidney | 37.6% |
| Adrenal | 6.7% |
| Pancreas | 0.7% |

Supplementary Table 4. HMC diagnostics for organ-specific injury model

| **Organ** | **Estimate (logit)** | **Est.Error** | **Rhat** | **Bulk_ESS** | **Tail_ESS** |
| --- | --- | --- | --- | --- | --- |
| Liver | -1.86 | 0.69 | 1.00 | 842 | 1168 |
| Spleen | -2.06 | 0.69 | 1.00 | 843 | 1141 |
| Bowel | -2.05 | 0.69 | 1.00 | 848 | 1156 |
| Kidney | -2.33 | 0.69 | 1.00 | 850 | 1172 |
| Adrenal | -3.32 | 0.69 | 1.00 | 859 | 1161 |
| Pancreas | -4.24 | 0.70 | 1.00 | 868 | 1185 |

Supplementary Table 5 Posterior Estimates for Interventions and Mortality in Pediatric Blunt Trauma

| **Outcome** | **Logit Estimate (Intercept)** | **Median Probability (%)** | **95% Credible Interval (%)** | **Between-Study SD (logit)** |
| --- | --- | --- | --- | --- |
| Intervention | -2.49 | 7.7 | 3.4 – 16.4 | 0.97 |
| Mortality | -4.25 | 1.4 | 0.5 – 3.9 | 1.45 |

Notes: Probabilities are back-transformed from the logit scale. Between-study SD reflects heterogeneity in study-level baseline risk.

Supplementary Table 6. Posterior Estimates from Bayesian Meta-Regression of Any Intra-Abdominal Injury

| **Parameter** | **Posterior Mean (%)** | **Posterior Median (%)** | **95% Credible Interval (%)** |
| --- | --- | --- | --- |
| Intercept | 32.3 | 24.8 | 1.5 – 90.9 |
| Age | 48.8 | 48.8 | 41.2 – 56.2 |
| Male proportion | 52.9 | 54.0 | 14.3 – 88.7 |

Supplementary Table 7 Grade assessment table

| **Outcome** | **Pooled Estimate (95% CrI / CI)** | **Studies (n)** | **Certainty of Evidence (GRADE)** | **Key Considerations** |
| --- | --- | --- | --- | --- |
| Any intra-abdominal injury | 84.5% (62–94%) | 15 | Moderate ⬤⬤⬤◯ | High heterogeneity; potential publication bias; Bayesian approach mitigates sparse data. |
| Liver injury | 12.7% (4.7–42.4%) | 15 | Low ⬤⬤◯◯ | Solid organ; moderate uncertainty; posterior probability >10% = 65%. |
| Spleen injury | 10.6% (3.9–37.8%) | 15 | Low ⬤⬤◯◯ | Posterior probability >10% = 55%; heterogeneity across cohorts. |
| Bowel injury | 10.7% (4.0–37.9%) | 15 | Low ⬤⬤◯◯ | Sparse events; moderate credible interval width. |
| Kidney injury | 8.3% (3.0–31.5%) | 15 | Low ⬤⬤◯◯ | Posterior probability >10% = 38%; lower prevalence. |
| Adrenal injury | 3.3% (1.1–14.3%) | 15 | Low ⬤⬤◯◯ | Rare outcome; limited data; wide CrI. |
| Pancreas injury | 1.3% (0.4–6.5%) | 15 | Low ⬤⬤◯◯ | Very rare; posterior probability >10% = 1%. |
| Interventions | 7.7% (3.4–16.4%) | 6 | Low ⬤⬤◯◯ | Small study number; heterogeneity present; stable posterior estimates. |
| Mortality | 1.4% (0.5–3.9%) | 8 | Low ⬤⬤◯◯ | Rare; credible intervals wide; robust posterior predictive checks. |
| Age (meta-regression) | 48.8% (41.2–56.2%) | 15 | Low ⬤⬤◯◯ | Suggests increased prevalence with older age; moderate uncertainty. |
| Male proportion (meta-regression) | 54.0% (14.3–88.7%) | 15 | Low ⬤⬤◯◯ | Higher prevalence with more male patients; wide credible interval. |
| Probability of injury by ISS subgroup | Low: 11% (2–35%); Moderate: 12% (1–49%); Severe: 12% (1–54%) | 12 | Low ⬤⬤◯◯ | Bayesian multilevel model with study-level random intercepts; overlapping credible intervals indicate no significant difference between ISS groups; highlights variability and uncertainty in predicting injury by ISS alone |

Supplementary Figure 1: Traffic plot of quality assessment


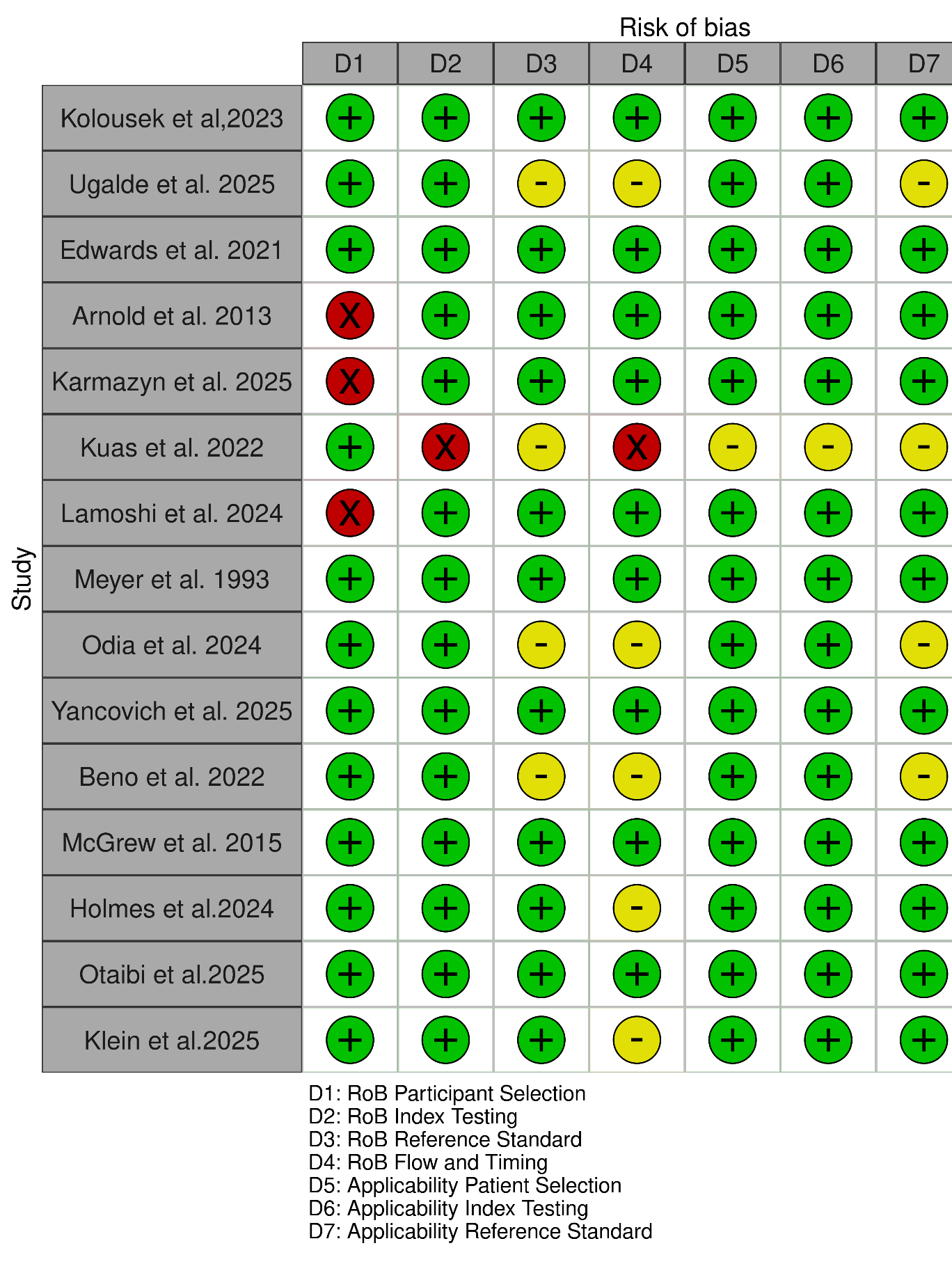


Supplementary Figure 2: Funnel plot shows asymmetry and outliers, indicating publication bias


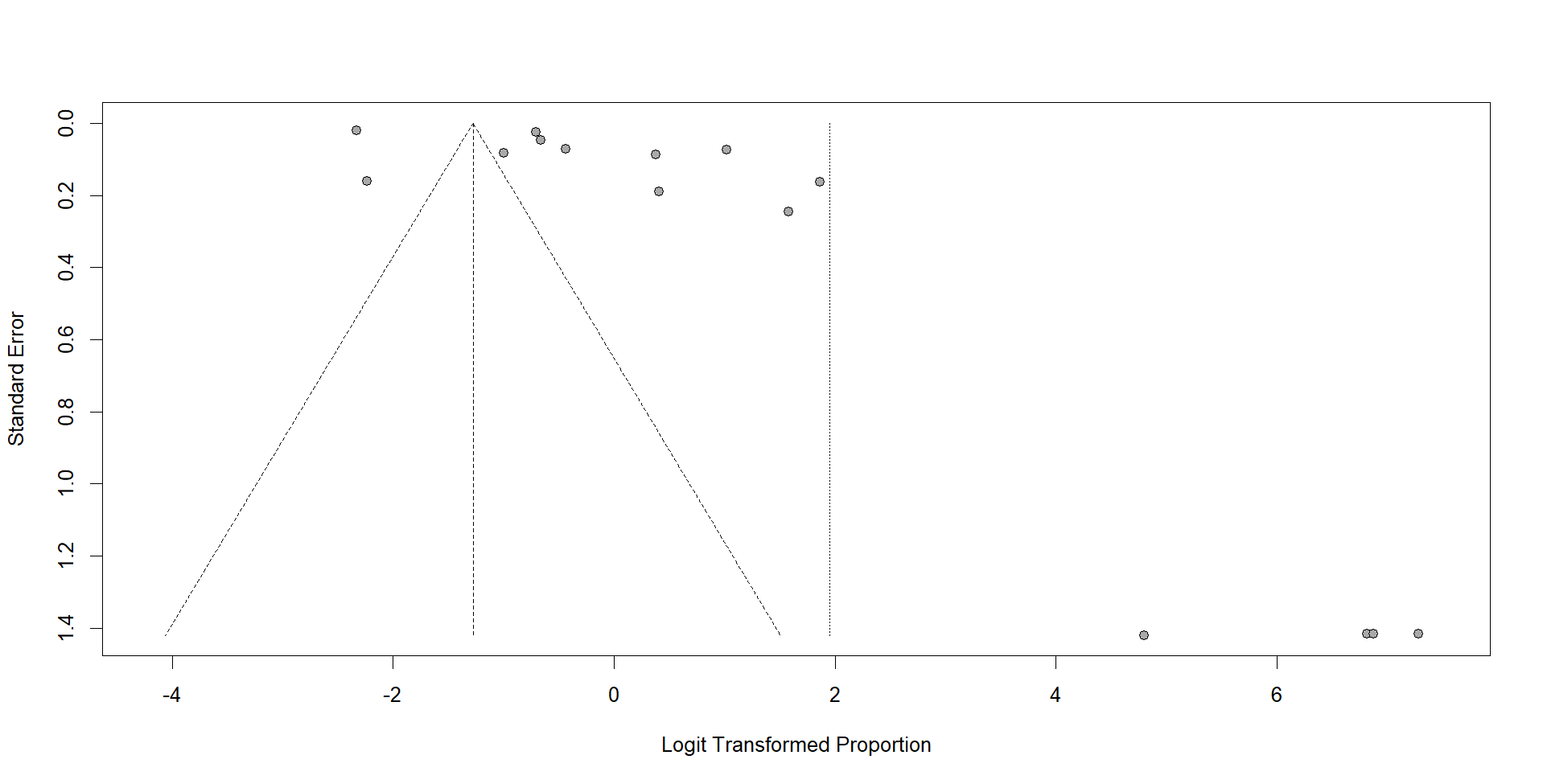

Supplement: Supplementary file 2 — Supplementary Material 2. [file 13049_2026_1578_MOESM2_ESM.docx]
